# Spatial-demographic analysis model for brain metastases distribution

**DOI:** 10.1007/s11547-025-01965-5

**Published:** 2025-02-28

**Authors:** Lin Zhang, Tongtong Che, Bowen Xin, Shuyu Li, Guanzhong Gong, Xiuying Wang

**Affiliations:** 1 The School of Computer Science, The University of Sydney, Sydney, NSW 2006 Australia; 2 State Key Laboratory of Cognitive Neuroscience and Learning, Beijing Normal University, Beijing, 100875 China; 3 Australian e-Health Research Centre, CSIRO, Sydney, NSW 2145 Australia; 4 Department of Radiation Physics, Shandong First Medical University Affiliated Cancer Hospital, Shandong Cancer Hospital and Institute, Jinan, 250117 China; 5 Department of Engineering Physics, Tsing Hua University, Beijing, 100084 China

**Keywords:** Brain metastases, Spatial relation, Distribution maps, Persistent homology

## Abstract

**Purpose:**

The distribution analysis of the morphologic characteristics and spatial relations among brain metastases (BMs) to guide screening and early diagnosis.

**Material and Methods:**

This retrospective study analysed 4314 BMs across 30 brain regions from MRIs of 304 patients. This paper proposed a unified analysis model based on persistent homology (PH) and graph modelling to provide a comprehensive portrait of BMs distribution. Spatial relationships are quantified through dynamic multiple-scale graphs constructed with Rips filtration. The multi-scale centrality importance and clustering coefficients are extracted to decode BMs spatial relations. Morphologic BMs characteristics are further analysed by varying radius and volume values that are considered as clinically influential factors. Finally, two-tailed proportional hypothesis testing is used for BM statistical distribution analysis.

**Results:**

For spatial analysis, results have shown a statistical increase in the proportions of high-level centrality BMs at the left cerebellum (p<0.01). BMs rapidly form graphs with high clustering rather than those with high centrality. For demographic analysis, the cerebellum and frontal are the top high-frequency areas of BMs with 0-4 and 5-10 radii. Statistical increases in the proportions of BMs at cerebellum (p<0.01).

**Conclusion:**

Results indicate that distributions of both BMs spatial relations and demographics are statistically non-random. This research offers novel insights into the BMs distribution analysis, providing physicians with the BMs demographic to guide screening and early diagnosis.

**Supplementary Information:**

The online version contains supplementary material available at 10.1007/s11547-025-01965-5.

## Introduction

Brain metastases (BMs) are the most common malignant brain tumours. BMs are a significant cause of patient morbidity and mortality and prevalent challenges in oncology and the common complications of cancer [1, 2]. The distribution analysis offers physicians a profound understanding of BMs [3–5]. Distributions of BM spatial relations and BM demographics contribute to understanding BMs. Firstly, in clinical consensus, the clustering growth of multiple BMs indicates short-term deterioration. The clustered BM distribution is a crucial basis for early screening and monitoring of treatment responses, especially for smaller tumours in the early stages, aiding in assessing the overall effectiveness of treatment. However, spatial relations are neglected by current feature engineering models. Secondly, several factors (including the location, size, and number of BMs) should be considered for optimal treatment [6]. Different tumour sizes respond differently to treatment, which affects treatment options [7]. Understanding the selective distribution is crucial for understanding BMs; nevertheless, comprehending the BM distribution remains incomplete.

Previous research primarily understands the distribution of BMs from three aspects: BM distributions of patients with different primary cancers [4, 8–10], BMs within specific brain regions [11–13], and BMs associated with multiple primary tumours across various brain regions [3, 5, 14]. Previous results show that the BMs non-randomly distribute throughout the brain, which has potential implications for therapeutic strategies and treatment outcomes [15]. Nevertheless, previous studies on BMs neglected the distribution of BMs with varying demographic values [16]. Furthermore, quantifying the spatial relations (e.g. clustering and centrality) within BMs and understanding the distribution of BMs with varying spatial relations assist with understanding the clustered tumours distribution. However, the analysis of spatial relations in BM distribution remains a gap. Overall, the distribution analysis of BM spatial relations and BM demographics are gaps in understanding BMs. Graphs are widely used to understand and analyse data structures and thus have the potential to qualify BM spatial relations and assist with understanding the distribution of BMs with varying spatial relations. Graph analytics includes techniques like centrality analysis, graph detection, and motif analysis. Centrality analysis aims to identify the most crucial nodes within a graph based on diverse criteria (e.g. degree centrality, betweenness centrality, and eigenvector centrality) [17]. However, quantifying a graph with optimal edges is challenging for multiple lesions without definite relations [18, 19].

This paper proposed a spatial-demographic distribution analysis model (SDDAM) to investigate the BM distributions from different spatial relations and morphologic values. For spatial relation analysis, this study proposed constructing dynamic multiple-scale graphs at varying distances and decoding the spatial relations in tumours based on PH and graph modelling. The model measures the spatial relations from the centrality importance and clustering coefficient. For demographic analysis, this research extracted radius and volume as morphologic features, measuring the size of BMs from different aspects. Radius and volume are unaffected by the image quality and easily observable, and treatment is different based on their size differences [7]. The brain is partitioned into 30 regions of interest (ROIs). In each ROI, two-tailed proportional hypothesis testing was utilised to compare the observed proportion with the random spatial distribution to assess the BM non-random distribution across the brain.

## Materials and methods

**Fig. 1:**
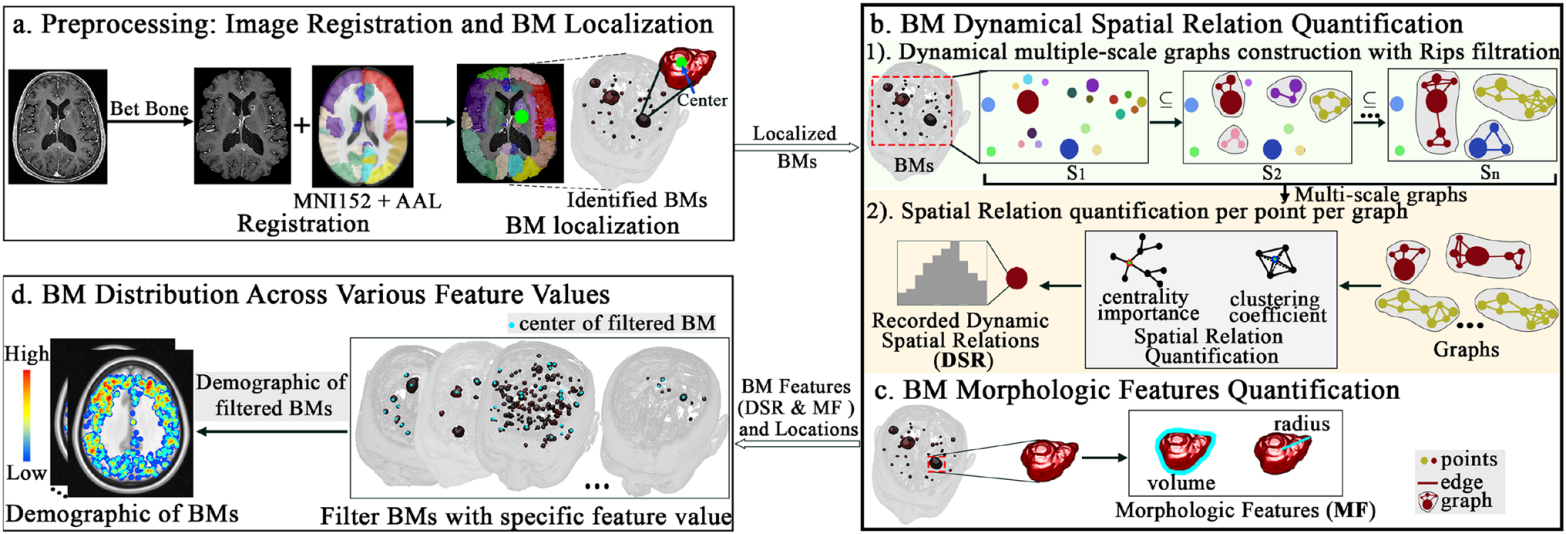
SDDAM overview: **a** The centres of BMs were taken as their locations. **b** Quantifying dynamical spatial relations of BMs (1) Constructing the dynamical multiple-scale graphs with Rips filtration. (2) Quantifying the spatial relation (centrality importance and clustering coefficient) per point within graphs. **c** Quantifying morphologic features of BMs. **d** BM distribution analysis for each feature quantified in steps **b** and **c**. S, scale; BM, brain metastases; MF, morphologic features; DSR, dynamic spatial relations

The proposed SDDAM includes three parts: preprocessing: image registration and BM localisation (Fig. 1a), feature quantification including BM dynamical spatial relation quantification (Fig. 1b) and BM morphologic features quantification (Fig. 1c), and BM distribution across various feature values (Fig. 1d).

### Dataset

**Fig. 2:**
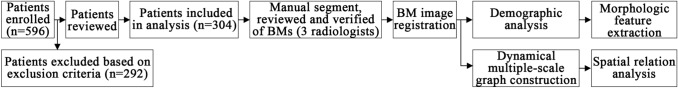
Study design diagram

**Fig. 3:**
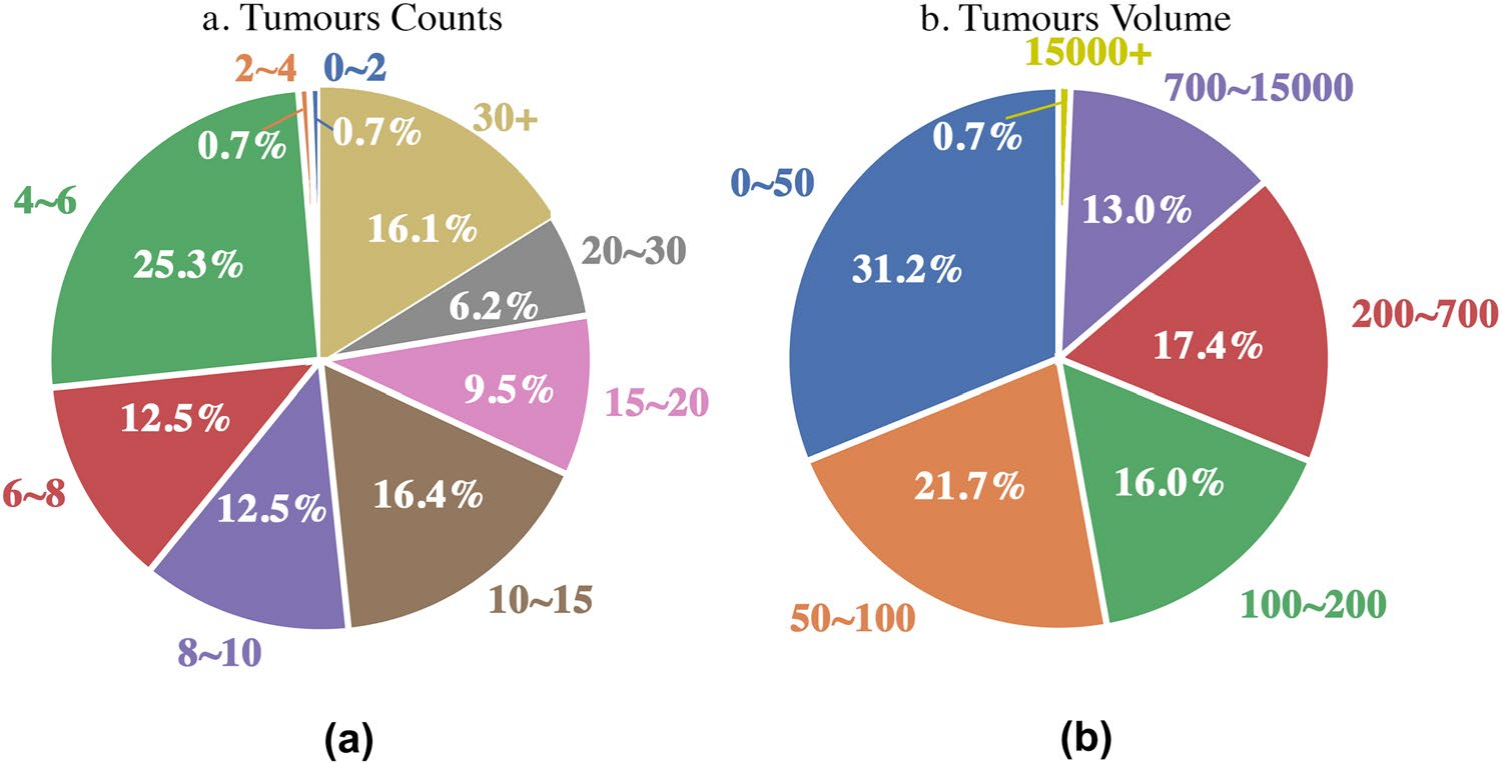
**a** Proportions of patients with different numbers of BMs. **b** Proportions of BMs with different volumes

We analysed 304 cases. In total, 596 patients were enrolled, whose MR images were scanned from the same scanner and with the same imaging standards. From this patient cohort, 292 patients were excluded if they had previously undergone surgical or radiotherapy treatment, had congenital malformations, or MRIs had severe artefacts. Three radiologists were involved in this research, including one conducted the screening and manual segmentation of BMs, and two senior radiologists then reviewed and verified all the results. When there was inconsistent evaluation among the three radiologists, consensus was reached through discussion. This research analysed 304 patients (including 4314 BMs) across 30 ROIs, including 155 males and 149 females. Among the primary tumours, there are cases of lung adenocarcinoma, small cell lung cancer, invasive ductal breast carcinoma, squamous cell lung cancer, ductal adenocarcinoma of the breast, glioblastoma, and others. All patients are to receive radiation therapy under the standard protocol established by the National Cancer Control Indicators (NCCI). There are 39 patients with diabetes; cholesterol levels are normal in all patients. Patient demographic information is listed in Table 1. The study design diagram is shown in Fig. 2. The mean number of BMs was 20 per patient. Figure 3a illustrates the ratio of patients with different numbers of tumours.

**Table 1:** Patient demographic information

Characteristic		No. of Patients (%)	No. of BM (%)
Primary tumour			
All patients		304 (100.00%)	4314 (100.00%)
Lung adenocarcinoma		175 (57.57%)	2458 (56.98%)
Small cell lung cancer		50 (16.45%)	481 (11.15%)
Lung cancer (others)		18 (5.92%)	339 (7.86%)
Invasive ductal breast carcinoma		13 (4.28%)	165 (3.82%)
Breast cancer (others)		8 (2.63%)	146 (3.38%)
Squamous cell lung cancer		7 (2.30%)	156 (3.62%)
Ductal adenocarcinoma of the breast		5 (1.64%)	234 (5.42%)
Glioblastoma		5 (1.64%)	34 (0.79%)
Others		23 (7.59%)	301 (6.98%)
Gender			
Male		155	
Female		149	
Age			
Mean(min–max)		58 (29–81)	
Diabetes			
Diabetes		39	
Non-diabetes		265	
Cholesterol			
Normal		304

### Preprocessing: image registration and BM localisation

*Image Registration:* This research used the MNI152 brain template image (https://fsl.fmrib.ox.ac.uk/fsl/fslwiki/Atlases) and the highly popular AAL brain atlas. The atlas was labelled into 30 key ROIs (as shown in Table 2). The rigid registration (using FSL [20]) and deformable registration (using symmetric normalisation in ANTs [21]) are performed to deform the MNI152 to patient images. For deformable registration, this research used cross-correlation (CC) with a sampling radius set to 4, and the flow standard deviation to smooth the gradient field was set to 3 [22]. Subsequently, the deformation fields are used to warp the AAL atlas accurately into the patient-specific anatomical space to correspond to tumorous regions in the AAL atlas identified. The standard template images are registered to the individual space, maintaining both its original topology and greyscale information of each patient image. *BM Localisation:* Due to variations in the size and shape of BMs, the corresponding three-dimensional centroids were determined to locate BMs [23].

### BM dynamical spatial relation quantification

#### Dynamical multiple-scale graphs construction with Rips filtration—Fig. 1b-1)

BMs are defined as a point cloud $P = \{p_1, p_2, p_i... p_n\}$. Each tumour is defined as a point $p_i$. A graph is represented as $G=(P, E)$, where *E* is the edge set $e \in E$. The PH generated by Rips filtration is widely used to capture spatial relations [24]. Rips filtration is the nested family of sub-graphs $\emptyset = K^0 \subseteq K^1 \subseteq ... \subseteq K^{s_n}$ for a range of scales $s_n\in {\mathbb {R}}$ from *P* [25]. The filtration process is illustrated in Fig. 1b-1), where $s_n$ are defined to capture the dynamic structural changes in BMs. In scale $s_i$, if the Euclidean distance *d* between two points (tumours) is less than the scale $d < s_i$, then points are connected as an edge *e* [26].

#### Structure relation quantification per point per graph—Fig. 1b-2)

A series of graphs are constructed with the increases in scale *s* from 10. The increase step is 5. In each scale $s_i$, the degree centrality [27] $dc_{p_u}^{s_i}$ and the eigenvector centrality [27] $ec_{p_u}^{s_i}$ represent the point importance and the neighbours importance of point $p_u$, respectively. The principal eigenvector is determined by calculating the adjacency matrix. The centrality importance ($ci_{p_u}^{s_i}$) of the point $p_u$ is calculated with the average of degree centrality and eigenvector centrality.

1 $$\begin{aligned} \begin{aligned}&dc_{p_u}^{s_i} = the\ number\ of\ neighbours \\&ec_{p_u}^{s_i} = \frac{1}{\lambda }\sum _{{p_v}\in P} A[{p_u}, {p_v}]ec_{p_v}^{s_i}, \forall {p_u} \in P \\&ci_{p_u}^{s_i} = \frac{dc_{p_u}^{s_i}+ec_{p_u}^{s_i}}{2} \end{aligned} \end{aligned}$$

where *A* is the adjacency matrix. If the points are connected, then $A[p_u,p_v]=1$; otherwise $A[{p_u},{p_v}]=0$. At scale $s_i$, the clustering coefficient [28] $cc_{p_u}^n$ measures the degree (*deg*) of the tumour $p_u$ in a graph that tends to cluster.

2 $$\begin{aligned} \begin{aligned} cc_{p_u}^{s_i} = \frac{2T({p_u}^{s_i})}{deg({p_u}^{s_i})(deg({p_u}^{s_i})-1)} \end{aligned} \end{aligned}$$

where $T({p_u}^{s_i})$ is the number of triangles through ${p_u}$, and $deg({p_u}^{s_i})$ is the degree of $p_u$.

The dynamic structural changes of the $p_u$ throughout multiple-scale are recorded by a set of *ci* and *cc*:

3 $$\begin{aligned} \begin{aligned} \{(ci_{p_u}^{s_i},cc_{p_u}^{s_i})\ s_i \in {\mathbb {R}}\} \end{aligned} \end{aligned}$$

### BM morphologic features quantification—Fig. 1c

The radius and volume for each BM are quantified as morphologic features, calculated with Pyradiomics [29]. Different radii and volumes group BMs. Due to the large number of BM with radii under 10 or volumes under 200, these are further subdivided into smaller aliquots.

### BM distribution across various feature values—Fig. 1d

Firstly, after feature extraction in the previous section, interested ranges are identified as the filtering threshold. Afterwards, BMs are filtered based on the filtering threshold (e.g. distribution of BMs with radii 0–2). The spatial relation values are between 0 and 1, which are divided into ten equal ranges. This research focused on the highest clustering coefficient and centrality importance, representing BMs tending to cluster together and the centre of the graph, respectively. Therefore, clustering coefficients exceeding 0.9 and centrality importance values exceeding 0.8 (no centrality importance higher than 0.9) are categorised as the filtering threshold. Following this, the proportion of filtered BMs in each region compared to the total number across all brain regions are calculated as the ratios of filtered BMs ${\hat{p}}$. The proportion of voxels in the ROI is defined as random distribution values $p_e$, since the occurrence risks of voxels with BMs are equal [4, 30]. To assess the BM non-random distribution throughout the brain, 2-tailed proportional hypothesis testing is employed to compare the observed occurrences of filtered BM ${\hat{p}}$ against the random distribution values $p_e$ for each ROI [31] as follows:

4 $$\begin{aligned} \begin{aligned} z = \frac{{\hat{p}}-p_e}{\sqrt{\frac{p_e(1-p_e)}{n}}} \end{aligned} \end{aligned}$$

where *n* shows the number of observed BMs. *z* represents the calculated z-score.

Bonferroni correction was utilised to adjust the significance level of multiple testing across all ROIs. The adjusted significance level for the *p*-value was determined by dividing the conventional threshold of 0.05 by the total number of ROIs (30), of which the statistical significance is *p* $\le$ 0.00167. A positive or negative z-score associated with ROI exhibiting statistically significant *p*-values indicates that the observed rate of BMs is significantly higher or lower than the random distribution rate, respectively [4, 30].

**Table 2:** Distribution property of BMs

Brain region	No. observed	Average radius (min - max)	Average volume (min - max)	Observed rate (%)	Random distribution Rate (%)	z-score	*p*-value
Cerebellum_L	555	3.53(1.12$-$18.56)	611.35(5.92$-$26783.59)	12.9%	6.0%	13.46	< 0.01
Frontal_R	437	3.69(1.10$-$18.88)	838.14(5.54$-$28177.09)	10.1%	9.2%	1.97	0.05
Frontal_L	383	3.49(1.10$-$19.21)	617.84(5.54$-$29671.14)	8.9%	9.0%	$-$0.36	0.72
Cerebellum_R	500	3.79(1.11$-$16.22)	715.39(5.67$-$17869.44)	11.6%	6.3%	10.96	< 0.01
Temporal_lobe_R	258	3.57(1.15$-$22.53)	847.53(6.35$-$47893.86)	6.0%	6.3%	$-$0.76	0.45
Temporal_lobe_L	238	3.68(1.23$-$19.89)	790.73(7.85$-$32958.92)	5.5%	5.8%	$-$0.93	0.35
Postcentral_Precentral_SuppMotorArea_L	246	3.65(1.25$-$18.68)	613.79(8.17$-$27290.76)	5.7%	5.9%	$-$0.67	0.50
Postcentral_Precentral_SuppMotorArea_R	198	3.72(1.07$-$19.34)	768.20(5.10$-$30279.59)	4.6%	5.7%	$-$3.53	< 0.01
Cuneus_Fusiform_Precuneus_L	148	3.61(1.46$-$13.69)	560.96(12.95$-$10742.34)	3.4%	4.0%	$-$2.09	0.04
Parietal_L	148	3.68(1.54$-$21.11)	722.65(15.44$-$39415.18)	3.4%	3.8%	$-$1.28	0.20
Occipital_L	188	3.87(1.21$-$18.28)	841.73(7.48$-$25592.20)	4.4%	5.5%	$-$3.50	< 0.01
Occipital_R	167	3.68(1.29$-$17.30)	555.08(9.00$-$21688.39)	3.9%	4.7%	$-$2.90	< 0.01
Parietal_R	169	3.78(1.25$-$15.46)	637.05(8.10$-$15491.09)	3.9%	4.0%	$-$0.23	0.82
Orb_Frontal_R	54	3.07(1.05$-$8.78)	222.17(4.92$-$2839.09)	1.3%	2.5%	$-$7.05	< 0.01
Cuneus_Fusiform_Precuneus_R	167	3.79(1.26$-$16.16)	557.28(8.48$-$17672.53)	3.9%	3.9%	$-$0.25	0.81
Hippocampus_R	72	3.47(1.33$-$12.17)	471.89(9.94$-$7556.10)	1.7%	2.4%	$-$3.73	< 0.01
Temporal_pole_R	41	2.73(1.56$-$6.92)	123.95(15.92$-$1389.07)	1.0%	1.4%	$-$2.90	< 0.01
Putamen_Pallidum_Caudate_R	33	2.88(1.79$-$10.66)	250.71(24.03$-$5074.96)	0.8%	1.3%	$-$3.86	< 0.01
Putamen_Pallidum_Caudate_L	51	3.28(1.78$-$11.84)	394.25(23.44$-$6957.25)	1.2%	1.2%	$-$0.27	0.79
Hippocampus_L	60	3.79(1.12$-$13.92)	722.45(5.85$-$11299.24)	1.4%	2.4%	$-$5.39	< 0.01
Orb_Frontal_L	51	3.30(1.09$-$12.02)	402.62(5.35$-$7275.56)	1.2%	2.3%	$-$7.03	< 0.01
Temporal_pole_L	28	3.17(1.60$-$7.01)	206.82(17.29$-$1441.32)	0.7%	1.1%	$-$3.76	< 0.01
Cingulum_Mid_R	26	3.28(1.43$-$12.24)	453.09(12.14$-$7680.08)	0.6%	1.2%	$-$5.02	< 0.01
Thalamus_R	28	2.94(1.48$-$8.08)	254.99(13.62$-$2205.58)	0.7%	0.6%	0.610	0.54
Cingulum_Post_L	0	nan(0.00$-$0.00)	nan(0.00$-$0.00)	0.0%	0.2%	-inf	0
Cingulum_Mid_L	14	3.75(1.85$-$7.65)	388.13(26.64$-$1877.70)	0.3%	0.9%	$-$7.06	< 0.01
Cingulum_Post_R	4	3.55(2.56$-$4.50)	208.65(69.96$-$382.84)	0.1%	0.2%	$-$1.86	0.06
Thalamus_L	21	3.29(1.81$-$13.94)	645.77(24.84$-$11341.60)	0.5%	0.6%	$-$1.02	0.31
Cingulum_Ant_L	18	3.56(1.47$-$9.59)	372.85(13.27$-$3694.17)	0.4%	0.7%	$-$3.14	< 0.01
Cingulum_Ant_R	11	2.51(1.68$-$3.24)	74.48(19.85$-$142.88)	0.3%	0.7%	$-$5.97	< 0.01

The *p*-value required for statistical significance was set at *p* $\le$ 0.00167 (0.05/30 ROI)

**Table 3:** Observed rate of BMs with spatial relations

	Clustering			Important		
	Observed rate (%)	z_score	*p*_value	Observed rate (%)	z_score	*p*_value
Cerebellum_L	19.9%	45.76	0	38.7%	3.74	<0.01
Frontal_R	9.8%	2.37	1.76E−02	0.0%	-inf	0
Frontal_L	8.4%	$-$2.89	3.82E−03	0.0%	-inf	0
Cerebellum_R	17.1%	37.91	0	25.8%	2.49	1.28E−02
Temporal_lobe_R	6.4%	0.50	0.62	0.0%	-inf	0
Temporal_lobe_L	5.0%	$-$5.04	< 0.01	0.0%	-inf	0
Postcentral_Precentral_SuppMotorArea_L	4.1%	$-$12.63	< 0.01	0.0%	-inf	0
Postcentral_Precentral_SuppMotorArea_R	2.7%	$-$24.02	< 0.01	0.0%	-inf	0
Cuneus_Fusiform_Precuneus_L	2.1%	$-$18.27	< 0.01	3.2%	$-$0.25	0.80
Parietal_L	2.5%	$-$10.39	< 0.01	0.0%	-inf	0
Occipital_L	4.0%	$-$9.56	< 0.01	0.0%	-inf	0
Occipital_R	4.1%	$-$4.01	< 0.01	0.0%	-inf	0
Parietal_R	2.7%	$-$10.87	< 0.01	0.0%	-inf	0
Orb_Frontal_R	1.8%	$-$6.93	< 0.01	0.0%	-inf	0
Cuneus_Fusiform_Precuneus_R	3.0%	$-$7.26	< 0.01	9.7%	1.08	0.28
Hippocampus_R	0.9%	$-$22.16	< 0.01	0.0%	-inf	0
Temporal_pole_R	0.9%	$-$7.14	< 0.01	0.0%	-inf	0
Putamen_Pallidum_Caudate_R	0.2%	$-$33.60	< 0.01	3.2%	0.61	0.54
Putamen_Pallidum_Caudate_L	0.6%	$-$11.34	< 0.01	9.7%	1.59	0.11
Hippocampus_L	0.7%	$-$25.86	< 0.01	0.0%	-inf	0
Orb_Frontal_L	1.2%	$-$13.94	< 0.01	0.0%	-inf	0
Temporal_pole_L	0.6%	$-$8.72	< 0.01	0.0%	-inf	0
Cingulum_Mid_R	0.4%	$-$17.97	< 0.01	6.5%	1.19	0.23
Thalamus_R	0.2%	$-$9.21	< 0.01	0.0%	-inf	0
Cingulum_Post_L	0.0%	-inf	0	0.0%	-inf	0
Cingulum_Mid_L	0.1%	$-$39.75	0	0.0%	-inf	0
Cingulum_Post_R	0.0%	$-$13.56	< 0.01	0.0%	-inf	0
Thalamus_L	0.2%	$-$11.23	< 0.01	3.2%	0.83	0.41
Cingulum_Ant_L	0.2%	$-$13.62	< 0.01	0.0%	-inf	0
Cingulum_Ant_R	0.5%	$-$4.52	< 0.01	0.0%	-inf	0

The observed rate of BMs with clustering coefficient exceeding 0.9 or centrality importance exceeding 0.8

## Results

### Proportions of BMs

**Fig. 4:**
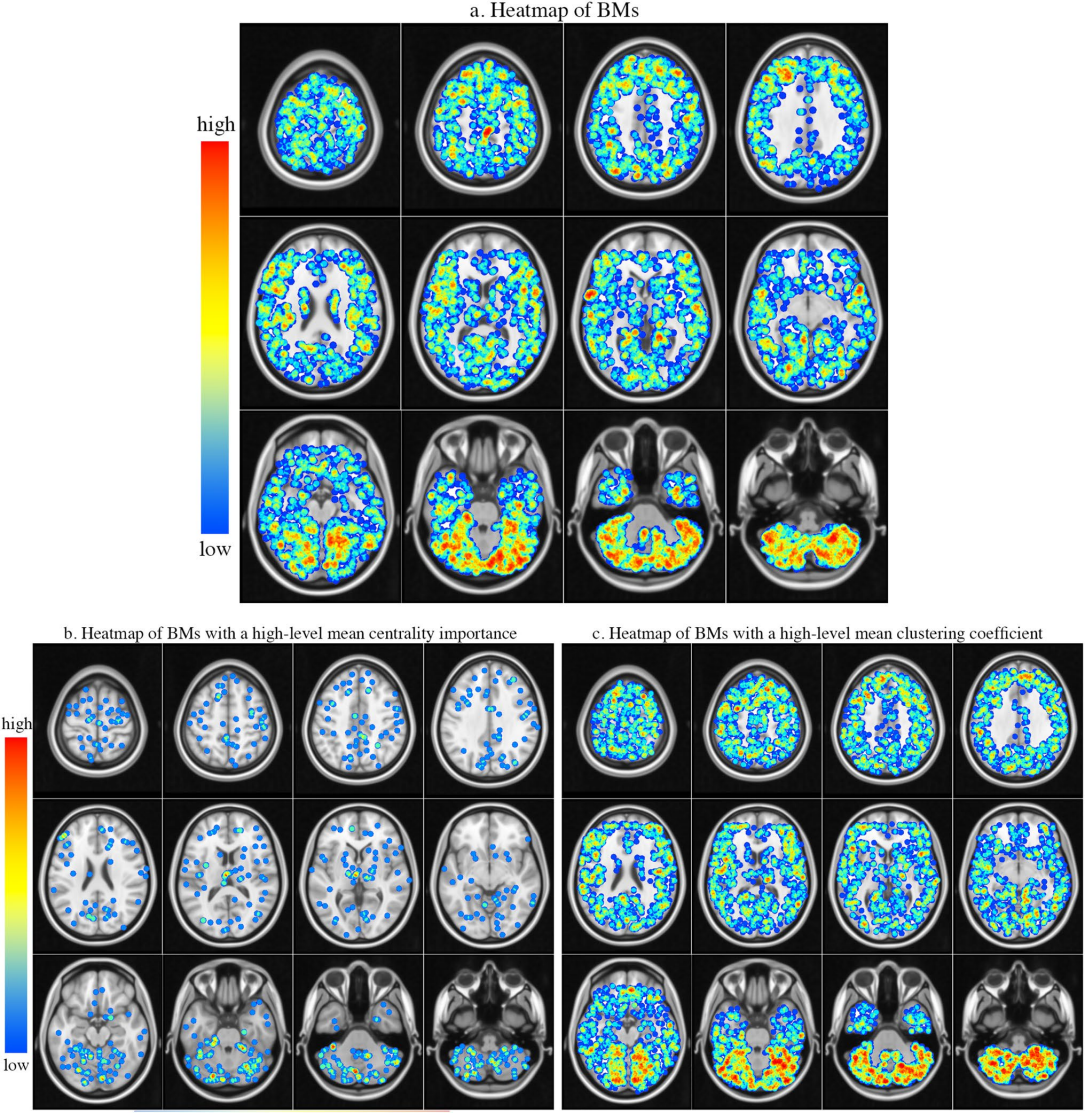
Axial image of the BM schematic distribution heatmap. A 5-mm-radius blue sphere was created to visualise the selected location, and spheres from all patients were overlaid to create the schematic distribution heatmap. In a, b, and c, the blue spheres represent the locations of BMs, and the locations of BMs with mean centrality importance and clustering coefficient values larger than 0.5, respectively. The distribution details shown in Table 2 and Fig. 5a and b

BMs are mostly located in the left cerebellum (12.9%), right cerebellum (11.6%), right frontal (10.1%), and left frontal (8.9%). No BMs are found in the left cingulum post. The left and right cerebellum display a statistically significant increase compared to the random distribution (*P* < 0.01). The summary of location, radius and volume for analysed BMs is shown in Table 2. Figure 4a illustrates the schematic BM distribution heatmap in the 30 ROIs. The generated heatmap is superimposed on the reference MNI152.

### BM distribution with different dynamical spatial relations

**Fig. 5:**
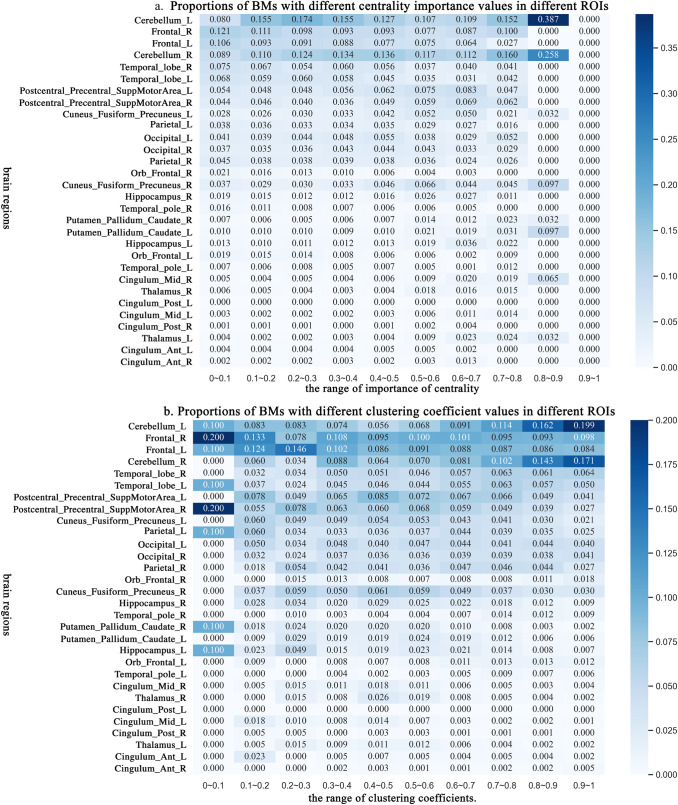

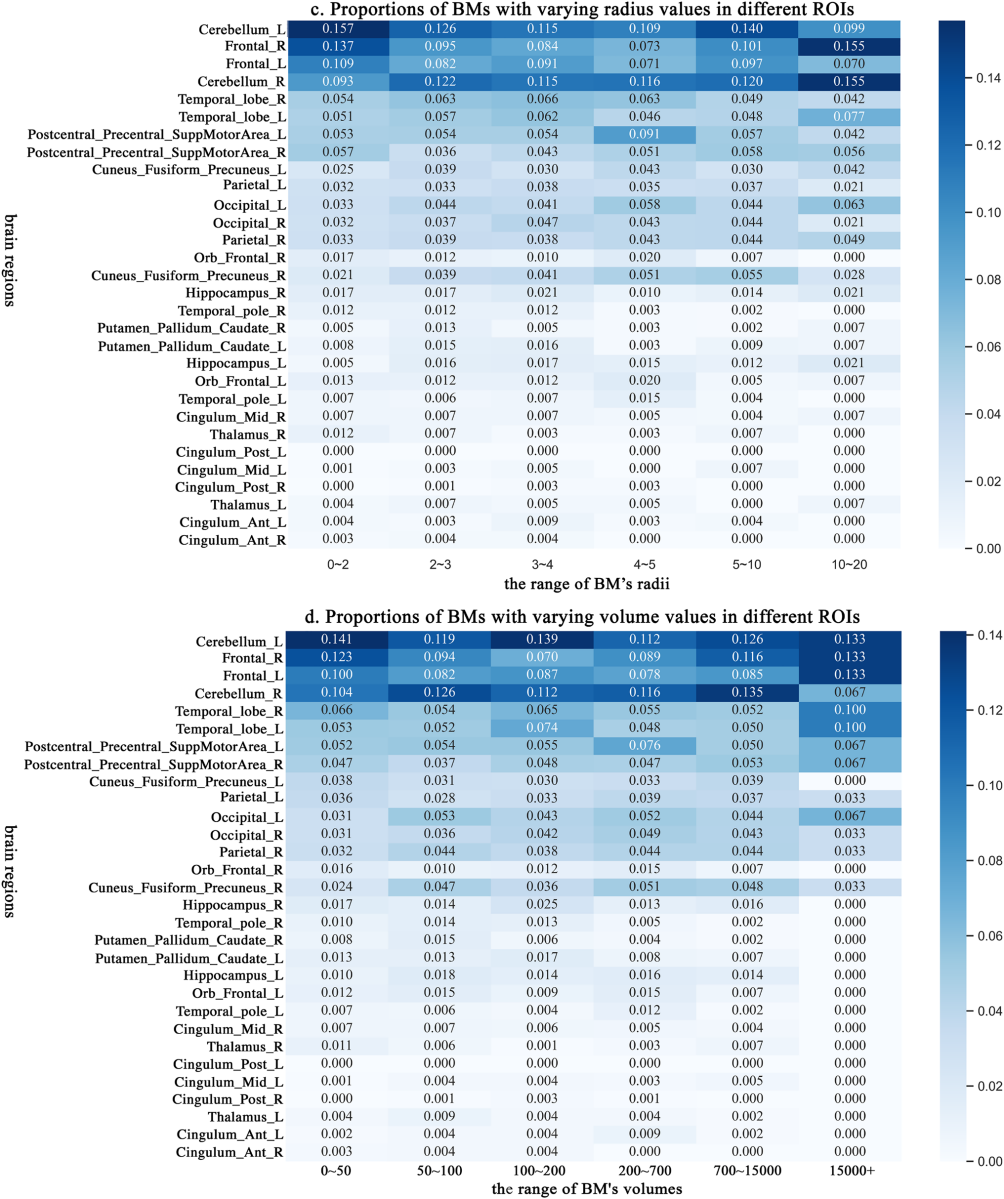
In different ROIs, **a** and **b** represent the proportions of BMs with different centrality importance and clustering coefficient values, respectively. **c** and **d** represent the proportions of BMs with varying radii and volume values, respectively

Figure 4b and c shows the schematic heatmap of the distribution of BMs with a mean value of clustering coefficient and centrality importance larger than 0.5 across multiple scales, respectively. Figure 5a and b details the distribution of BMs with varying spatial relations. Moreover, Table 3 illustrates the distribution of BM with high-level spatial relations.

#### Proportions of BMs with different centrality importance values

*Distribution values:* Approximately, 64.5% of BMs with centrality importance ranging from 0.8 to 0.9 are located in the cerebellum (left and right). Moreover, 31.2% of BMs with centrality importance between 0.7 and 0.8, as well as 22.1% of BMs with centrality importance ranging from 0.6 to 0.7, are situated in the cerebellum (left and right). *Statistical analysis:* The left cerebellum presents a statistically significant increase (P<0.01) in proportion for the BMs with high-level centrality importance than random distribution.

#### Proportions of BMs with different clustering coefficient

*Distribution values:* Around 37% of BMs with clustering coefficients exceeding 0.9 are located in the cerebellum (left and right). The left and right of cerebellum and frontal are the top-frequency areas for BMs with clustering coefficients exceeding 0.6. *Statistical analysis:* The left and right cerebellum show a statistically significant increase in BM with high-level clustering coefficient than random distribution.

#### Case study

**Fig. 6:**
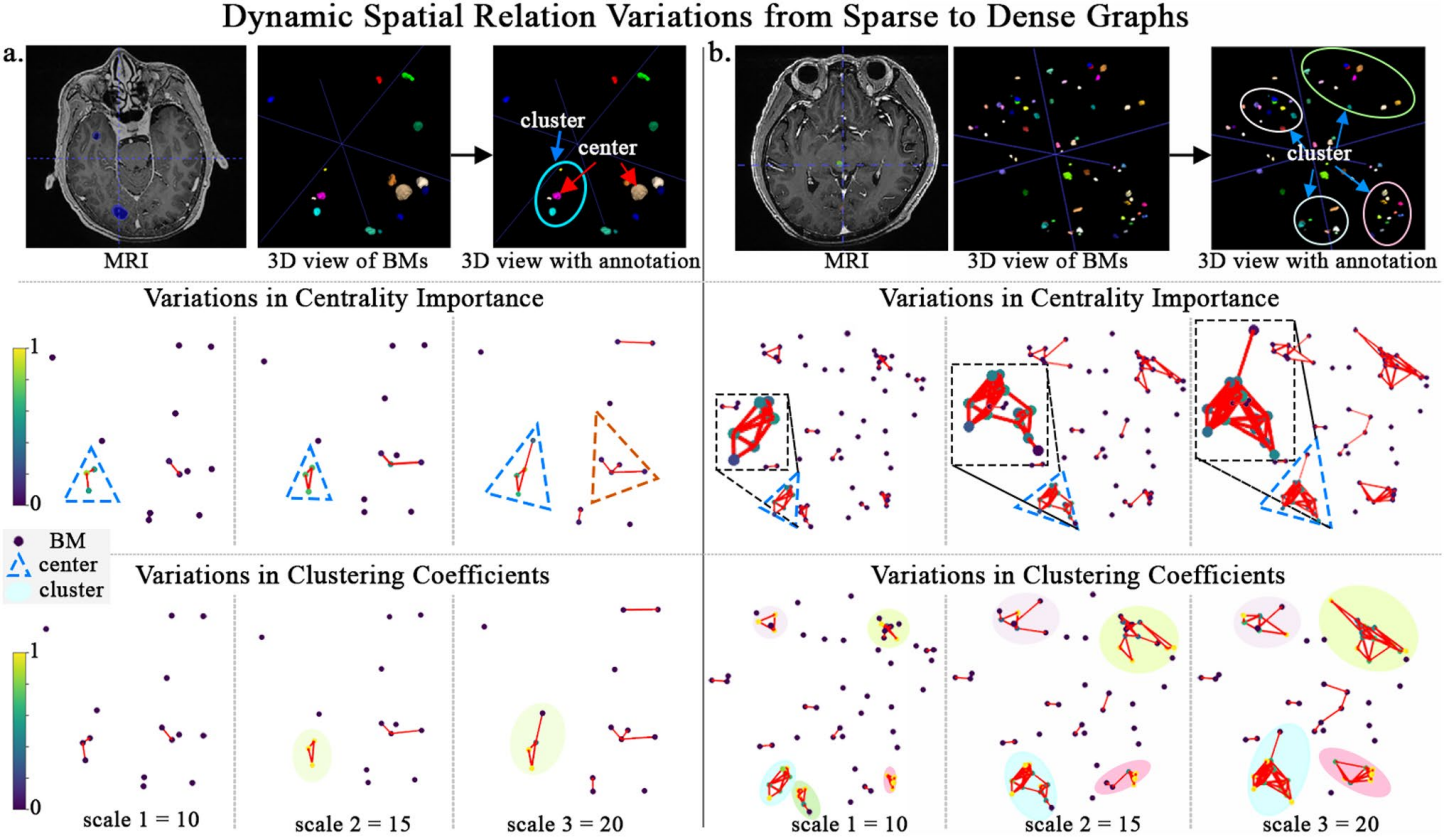
Cases illustrate dynamic spatial relation variations from sparse to dense graphs. Cases a and b demonstrate a small and larger quantity of BMs, respectively. The node in each graph represents a BM in the MRI. The darker and lighter colours represent low- and high-centrality importance or clustering coefficients, respectively. The oval represents the cluster, and the dotted triangle represents the community containing nodes of high-central importance

Cases in Fig. 6 illustrate dynamic spatial relation variations from sparse to dense (include scale 10, 15 and 20) graphs. The first row shows the MRI, three-dimensional images, and the three-dimensional image annotated cluster and centre BM. The following two rows represent the variations of centrality importance and clustering coefficient in dynamical graphs. Cases a and b have a small and large number of BMs, respectively. In scale 10, BMs in case a started to connect and BMs in case b formed clusters. Clusters started forming in case a at scale 15. At scale 20, case a formed two communities, each containing nodes with high-central importance. The node within the blue triangle has greater central importance than the one within the orange triangle due to more connections within the community. Moreover, the four clusters in case b became more compact at scale 20. These cases illustrate how centrality and clustering in BMs are gradually revealed in the dynamic graph from a sparse to dense connection.

### BM distribution with different morphologic feature

Figure 5c, d details the distribution of tumours with varying radii and volumes, respectively.

#### Proportions of BMs with different radii

The left and right of cerebellum and frontal are the top high-frequency areas of BMs with 0–4 and 5–10 radii. The left cerebellum is the highest frequency area of BMs with 0–4 and 5–10 radii. The frequency of BMs with 0–20 radii in cingulum ant (left and right), cingulum post (left and right), left thalamus, and left cingulum mid is less than 1%.

#### Proportions of BMs with different volumes

The largest and smallest volumes of BMs are 47893 and 4, respectively. The average volume is 650. More details are given in Fig. 3b. The left and right of cerebellum and frontal are the four top-frequency areas of BMs with volume under 15000. In these four areas, 46.8%, 42.1%, 40.8%, and 39.5% of BMs have volumes in 0-50, 50-100, 100-200, and 200-700, respectively.

## Discussion

Firstly, the proposed SDDAM will be compared with current research. Then, the distinct distribution patterns of BMs within the brain will be investigated. After that, an overview of the ROI with a high proportion of BMs will be provided. Lastly, we investigate the relationship between primary tumours and the distribution of BMs.

### Contrasting Spatial-Demographic Model with Current BM Analysis

For the demographic analysis, currently, BM distribution is investigated in three key areas: different primary cancers, specific brain regions, and multiple primary tumours across various ROIs. Specifically, distribution research primarily focused on the locations and sizes, with particular attention on patients with different primary cancers, such as small cell lung cancer [4], lung cancer [8] and breast cancer [9, 10]. Some studies explored the distribution within hippocampus [11–13], and BMs associated with multiple primary tumours across various ROIs [3, 5, 14]. This research expands on current BM demographic models by investigating BM distributions through various spatial relations and morphologic values. The results show that BMs are frequently in the cerebellum and frontal and less present in the cingulum and thalamus, which confirms findings from past reports [3–5]. Notably, the proposed SDDAM innovatively investigates the demographic of BM with varying spatial relations and morphologic values. Further discussion will follow.

### BMs tend to form small and tight graphs instead of high-centrality

BMs exhibit distinct distribution patterns within the brain. Understanding these spatial tendencies could give physicians critical insights and help avoid overlooking tumours. Dynamic graphs are constructed to fully present the structure of BMs across multiple scales, not just one specific structure. High-centrality importance BMs are characterised by extensive connections with significant neighbouring entities. Meanwhile, high clustering coefficients indicate BMs within a graph where members exhibit closeness. Figure 6 intuitively demonstrates the variation of centrality importance and clustering coefficients in the case with smaller and larger quantity BM at scales 10, 15 and 20. Clusters are already formed at small scales in these cases.

**Fig. 7:**
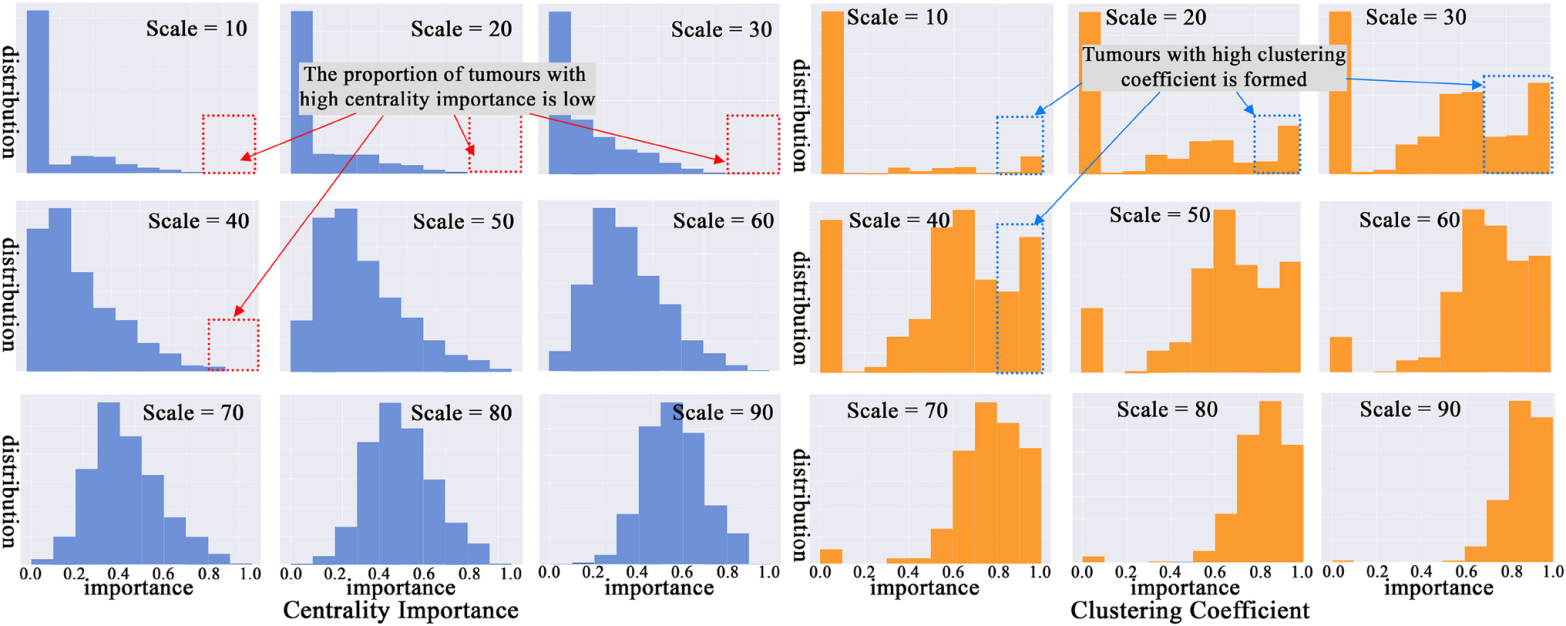
Comparison of the distribution for centrality importance (left) and clustering coefficients (right)

Figure 7 illustrates the comparison of the changes in distributions for centrality importance (left) and clustering coefficients (right) across scales 10–90. The x-axis represents the value range, with higher values on the right. Taller bars indicate higher proportions. Figure 7 shows, at small scales (e.g. scale = 10, 20, 30, 40), the proportion of high clustering coefficient BMs is higher than high-centrality importance BMs. This indicates that small, tightly connected graphs have formed at a small scale rather than graphs with high-centrality values. This is also observed in case study b (Fig. 6b), where four clusters form at scale 10, with unobvious node centrality importance. As the scale increases, more BMs with high centrality are shown, indicating the growth of tightly connected graphs. The results show that BMs quickly form some small graphs with higher clustering than with high-centrality tumours. Radiologists should diligently search for small tumours near existing ones to minimise the risk of missing lesions.

### Overview of brain regions with a high proportion of BMs

BMs distribute non-stochastically across brain regions. BMs with varying features present some similar and different distributions. As discussed before, physicians may gain insights into tumours through spatial patterns. Therefore, this section will further explore brain regions with a high BM (varying features) proportion, offering physicians a clearer overview of ROIs. BMs are mainly located at left cerebellum (12.9%), right cerebellum (11.6%), right frontal (10.1%), and left frontal (8.9%). These regions are the top high-frequency area of BMs with 0–4 and 5–10 radii and BMs with volume under 15000. The left cerebellum exhibits a statistically significant increase in both the number of BMs and BMs with high clustering coefficient or centrality importance. Conversely, the left frontal region demonstrates a statistically significant decrease in BMs with high-level clustering coefficient.

### Relationship between primary tumour and the distribution of BMs

**Fig. 8:**
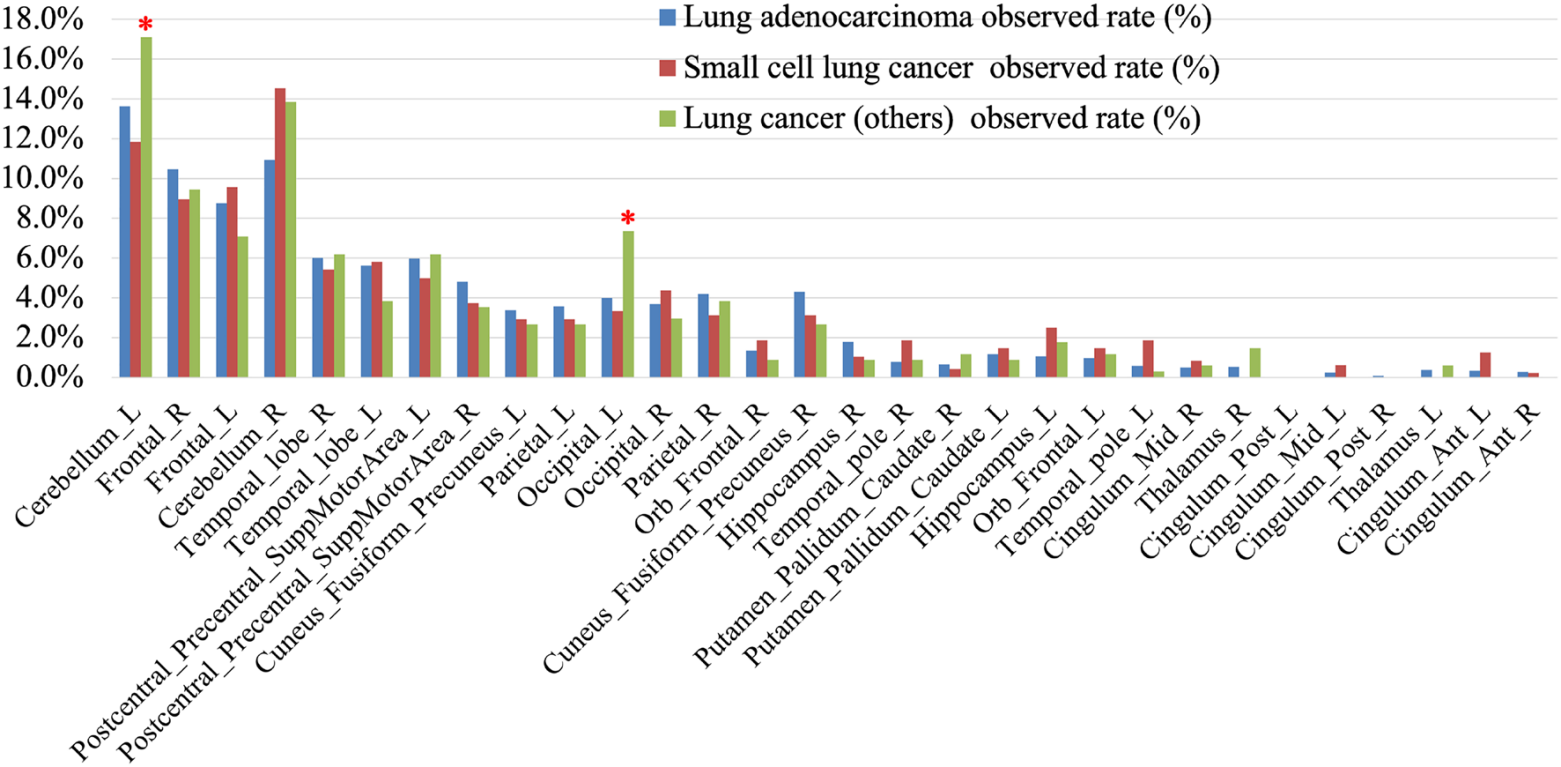
Comparison of observed rate between BMs with different primary tumours in various brain regions. *Highlights that in comparison with its lowest quantity of lung cancers (others), its tumour proportion is the highest in the left cerebellum and left occipital among the three primary tumours

In this research, we preliminarily focused on analysing the relationship between the primary tumours of lung adenocarcinoma, small cell lung cancer, and lung cancers (others) and the distribution of BMs due to the limited samples of other primary tumours. Figure 8 compares the distribution difference of BMs with different primary tumours. The results show that BM with these three primary tumours were most likely to occur in the left and right of cerebellum and frontal. Lung adenocarcinoma BMs and small cell lung cancer BMs have the highest probability of BMs in the left cerebellum (13.6%) and right cerebellum (14.6%), respectively. The quantity of lung cancers (others) is the lowest, but its tumour proportion in the left cerebellum (17.1%) and left occipital (7.5%) is the highest among the three primary tumours. Detailed proportion values are listed in Table S.1. Table 1 illustrates the distribution of patients and BMs with different primary tumours. There are 5.42% of BMs with ductal adenocarcinoma of the breast, which is much higher than the proportion of patients with ductal adenocarcinoma of the breast (1.64%).

## Limitation and future works

This research mainly focused on developing the unified analysis model based on persistent homology (PH) and graph modelling to provide a comprehensive portrait of BM distribution. In our future work, we plan to investigate the spatial relations for BMs with the same type of primary tumours, relations between primary tumours and the spatial structures of BMs, and the spatial dynamic changes of BMs in multiple time points by conducting collaborative research between multiple medical institutions to collect a large amount of data.

## Conclusion

The SDDAM was proposed to investigate the BM distribution from varying spatial relations and morphologic feature values. Spatial relations within BMs are proposed to be quantified based on PH and graph modelling. Morphologic features are quantified with radii and volumes, which clinically influence BM outcomes. Two-tailed proportional hypothesis testing was employed to compare the observed BM proportion and the random BM distribution proportion. Results show that the left and right of the cerebellum and frontal lobes are the top-frequency areas for BMs. They also display a high proportion of BMs with high-level centrality importance and clustering coefficients. BMs are likelier to form clustered graphs in small areas than high-centrality graphs. This research offers new insights into BM distribution analysis. It provides a deep understanding of spatial relations and morphologic distribution of BMs, guiding screening and early diagnosis.

## Supplementary Information

Below is the link to the electronic supplementary material.

Supplementary file 1 (docx 1115 KB)
