# Supplementary material for: Spatial-demographic analysis model for brain metastases distribution
Source: Radiol Med. 2025 Feb 28;130(3):397–411. doi: 10.1007/s11547-025-01965-5 (PMC11903647; doi:10.1007/s11547-025-01965-5)
Supplement: Supplementary file 1 — (docx 1115 KB) [file 11547_2025_1965_MOESM1_ESM.docx]

To examine prognostic value of our research. The experiment and analysis are conducted on both our data and extra multiple-time-point public data [1] for assessing the prognostic values of the spatial patterns as identified in our research when predicting the important tumour changes at a proceeding time interval. Firstly, previously validated prognostic features [2,3] are extracted from our and public datasets respectively; Then, the two datasets are correspondingly classified into two groups respectively: (1) the dense and sparse groups of BMs are formed from our dataset based on our finding of cluster spatial patterns, and (2) feature-increasing and feature-decreasing groups of BMs are formed from public dataset based on changes in prognostic features at the subsequent time point; Finally, U Test is performed to statistically analyse the correlations of the prognostic features and spatial relation characteristics (as identified by our research).

Firstly, S.1a shows the statistical analysis results of testing the discriminative capacities of (1) our spatial pattern features extracted from our in-house dataset, and (2) reported prognostic feature from the public data, when classifying the BMs into different group respectively. The results indicate our finding (cluster spatial pattern) is effective for differentiating BMs into different (dense and sparse) groups. S.1b and S.1c present statistical analyses of the sparse and dense groups against feature-increasing and feature-decreasing groups, respectively. As illustrated in S.1b, the results show differences between the sparse group and feature-increasing group, and S.1c illustrates differences between the dense group and feature-decreasing group. These results show that BMs classified by our identified cluster spatial patterns statistically correlate with prognostic features in tumour changes, which hence indicates the prognostic value of our research finding.

S.1. Statistical analysis results of shape elongation on both our data and extra multiple-time-point public data.


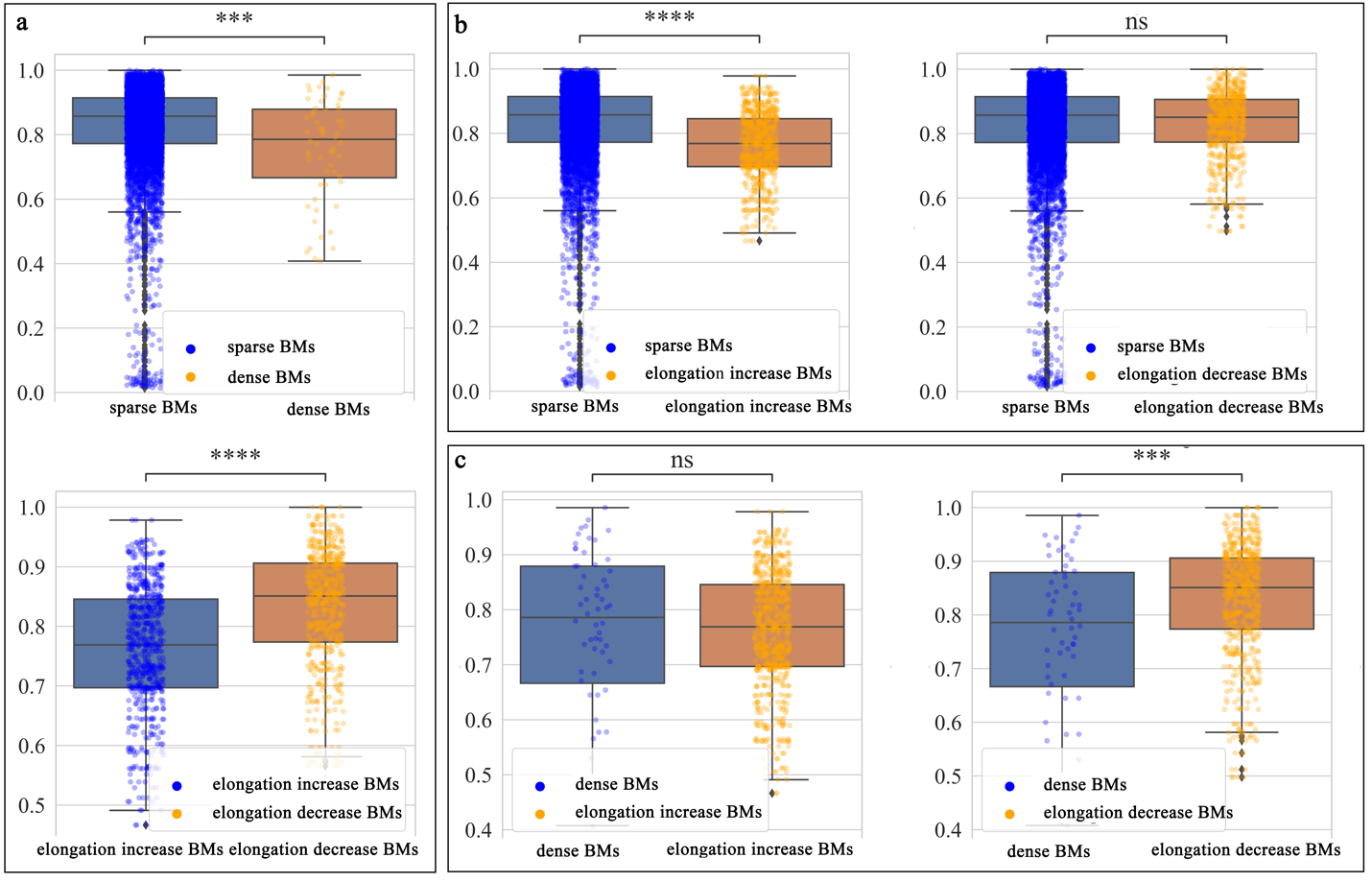


| Table S.1 Observe Rate of BMs with different primary tumour in various brain regions. | | | | |
| --- | --- | --- | --- | --- |
|  | Observed Rate (%) | | | |
|  | Lung adenocarcinoma 175 patients | Small cell lung cancer  50 patients | | Lung cancer (Others)  18 patients |
| Cerebellum_L | 13.6% | 11.9% | **17.1%** | |
| Frontal_R | 10.5% | 8.9% | 9.4% | |
| Frontal_L | 8.8% | 9.6% | 7.1% | |
| Cerebellum_R | 10.9% | 14.6% | 13.9% | |
| Temporal_lobe_R | 6.0% | 5.4% | 6.2% | |
| Temporal_lobe_L | 5.6% | 5.8% | 3.8% | |
| Postcentral_Precentral_SuppMotorArea_L | 6.0% | 5.0% | 6.2% | |
| Postcentral_Precentral_SuppMotorArea_R | 4.8% | 3.7% | 3.5% | |
| Cuneus_Fusiform_Precuneus_L | 3.4% | 2.9% | 2.7% | |
| Parietal_L | 3.6% | 2.9% | 2.7% | |
| Occipital_L | 4.0% | 3.3% | **7.4%** | |
| Occipital_R | 3.7% | 4.4% | 3.0% | |
| Parietal_R | 4.2% | 3.1% | 3.8% | |
| Orb_Frontal_R | 1.3% | 1.9% | 0.9% | |
| Cuneus_Fusiform_Precuneus_R | 4.3% | 3.1% | 2.7% | |
| Hippocampus_R | 1.8% | 1.0% | 0.9% | |
| Temporal_pole_R | 0.8% | 1.9% | 0.9% | |
| Putamen_Pallidum_Caudate_R | 0.7% | 0.4% | 1.2% | |
| Putamen_Pallidum_Caudate_L | 1.2% | 1.5% | 0.9% | |
| Hippocampus_L | 1.1% | 2.5% | 1.8% | |
| Orb_Frontal_L | 1.0% | 1.5% | 1.2% | |
| Temporal_pole_L | 0.6% | 1.9% | 0.3% | |
| Cingulum_Mid_R | 0.5% | 0.8% | 0.6% | |
| Thalamus_R | 0.5% | 0.0% | 1.5% | |
| Cingulum_Post_L | 0.0% | 0.0% | 0.0% | |
| Cingulum_Mid_L | 0.2% | 0.6% | 0.0% | |
| Cingulum_Post_R | 0.1% | 0.0% | 0.0% | |
| Thalamus_L | 0.4% | 0.0% | 0.6% | |
| Cingulum_Ant_L | 0.3% | 1.3% | 0.0% | |
| Cingulum_Ant_R | 0.3% | 0.2% | 0.0% | |

**Reference**

[1] Ocaña-Tienda, B., Pérez-Beteta, J., Villanueva-García, J. D., Romero-Rosales, J. A., Molina-García, D., Suter, Y., et al. (2023). A comprehensive dataset of annotated brain metastasis MR images with clinical and radiomic data. *Scientific data*, *10*(1), 208.

[2] Jaberipour, M., Soliman, H., Sahgal, A., & Sadeghi-Naini, A. (2021). A priori prediction of local failure in brain metastasis after hypo-fractionated stereotactic radiotherapy using quantitative MRI and machine learning. *Scientific Reports*, *11*(1), 21620.

[3] Mouraviev, A., Detsky, J., Sahgal, A., Ruschin, M., Lee, Y. K., Karam, I., et al. (2020). Use of radiomics for the prediction of local control of brain metastases after stereotactic radiosurgery. *Neuro-oncology*, *22*(6), 797-805.
